# Consuming iodine enriched eggs to solve the iodine deficiency endemic for remote areas in Thailand

**DOI:** 10.1186/1475-2891-9-68

**Published:** 2010-12-20

**Authors:** Wiyada Charoensiriwatana, Pongsant Srijantr, Punthip Teeyapant, Jintana Wongvilairattana

**Affiliations:** 1 Department of Medical Sciences, Ministry of Public Health, Nonthaburi, Thailand; 2 Department of Soil Science, Kasetsart University, Nakhon Pathom, Thailand

## Abstract

**Background:**

Evidence showed that the occurrence of iodine deficiency endemic areas has been found in every provinces of Thailand. Thus, a new pilot programme for elimination of iodine deficiency endemic areas at the community level was designed in 2008 by integrating the concept of Sufficient Economic life style with the iodine biofortification of nutrients for community consumption.

**Methods:**

A model of community hen egg farm was selected at an iodine deficiency endemic area in North Eastern part of Thailand. The process for the preparation of high content iodine enriched hen food was demonstrated to the farm owner with technical transfer in order to ensure the sustainability in the long term for the community. The iodine content of the produced iodine enriched hen eggs were determined and the iodine status of volunteers who consumed the iodine enriched hen eggs were monitored by using urine iodine excretion before and after the implement of iodine enrichment in the model farm.

**Results:**

The content of iodine in eggs from the model farm were 93.57 μg per egg for the weight of 55 - 60 g egg and 97.76 μg for the weight of 60 - 65 g egg. The biological active iodo-organic compounds in eggs were tested by determination of the base-line urine iodine of the volunteer villagers before and after consuming a hard boiled iodine enriched egg per volunteer at breakfast for five days continuous period in 59 volunteers of Ban Kew village, and 65 volunteers of Ban Nong Nok Kean village. The median base-line urine iodine level of the volunteers in these two villages before consuming eggs were 7.00 and 7.04 μg/dL respectively. After consuming iodine enriched eggs, the median urine iodine were raised to the optimal level at 20.76 μg/dL for Ban Kew and 13.95 μg/dL for Ban Nong Nok Kean.

**Conclusions:**

The strategic programme for iodine enrichment in the food chain with biological iodo-organic compound from animal origins can be an alternative method to fortify iodine in the diet for Iodine Deficiency Endemic Areas at the community level in Thailand.

## Background

Iodine deficiency disorders (IDD) had been widely recognized as one of the important public health problems especially in developing countries throughout the world [1]. Thailand, one of the developing countries in South East Asia has started the public health activities on elimination of IDD endemic areas since 1989. In 1991, a national survey on total goitre prevalence (TGP) in 20,596 schools from 3,366,867 children was done in 53 provinces throughout the country with the mean TGP of 15.79% [2]. Later in 1994, World Health Organization (WHO) produced a document in collaboration with United Nations International Children's Emergency Fund (UNICEF) and International Council for Control of Iodine Deficiency Disorders (ICCIDD) for the guidance concerning the IDD surveillance indicators and salt iodization has been selected as a strategy to control and elimination of IDD [3]. Since then Thailand has emphasized various kind of strategic planning for elimination of IDD endemic areas such as iodization of drinking water for villagers, administration of iodine capsules every 6-10 months for population in remote areas where transportation was the main problem for the enrichment of iodine in communities, iodization of edible salt at 30 ppm for daily use in household etc. In 2006, a survey for the use of iodine salts from 819 households were done in iodine deficiency endemic areas in Udon Thani province and found that only 10.26% of the households consumed iodine edible salts which were passed the standard of edible iodized salts (not less than 30 ppm of iodine) declared by Thailand FDA [4]. In addition, the TSH index for monitoring IDD by WHO/UNICEF/ICCIDD guideline, showed that during the years 2003 - 2006 the number of neonates having TSH >5 mU/L were 13.54%, 15.28%, 21.55%, 19.56% respectively [5]. These TSH index showed that Thailand was exposed to iodine deficiency condition and also indicated that the current public health activities did not achieve the goal for the elimination of IDD.

A new pilot programme has been proposed to public health sector to introduce a more stable form of iodine in daily nutrition through the existence of biological active iodo-organic compounds from animal and plant origins in the natural food chain which will be sustainable in the long-term. The design of the programme was initiated with the aim to increase the content of iodine in eggs and vegetables. National Statistic Data on Thailand eggs consumption during 2006 and 2007 were 9,789 and 9,376 million eggs respectively or 142 and 150 eggs per person per year [6]. Thus, the hen eggs would be one of the reliable sources for iodine consumption in daily nutrition. This new programme would be implemented with the philosophy of Sufficient Economy Concept introduced by His Majesty the King of Thailand to conduct all Thai peoples living for better quality of life with the appropriate needs of socio-economic development in moderation, reasonableness and self-immunity for sufficient protection from both internal and external impacts arising with knowledge and spirit to supply their long-term necessities throughout the country starting from the level of the families, communities as well as the nation. It was believed that with this new concept of the biological active iodo-organic compound enrichment in the natural food chain through the Sufficient Economy Philosophy, the new programme for elimination of iodine deficiency endemic areas would be successfully implemented through the cooperation with all concerned sectors throughout the country.

## Methods

The design of the study was to determine the iodine status of communities by using urine iodine excretion concept before and after the implement of iodine enrichment in the natural food chain. The collecting of 858 urine of child bearing age women as first morning mid-stream urine from 5 districts in Udon Thani province were done in 2006 in order to establish base-line iodine status of the areas before implementing the programme. The urine specimens were transported to the laboratory immediately in ice boxes and kept frozen at -20°C until the analysis. The urine iodine was determined by Inductively Coupled Plasma Mass Spectrometry (ICP-MS) with the use of tellurium as internal standard. During the assay performance, the internal quality control samples prepared from urine iodine reference standard code 2670 from US-National Institute of Standards and Technology (NIST) were inserted in every ten samples of the running assay [7,8].

In 2008, the model programme was operated in cooperation with Napu Sub-district Municipality and the experimental design was approved from the Napu Sub-district Municipal Committee in compliance with the Helsinki Declaration. The propose programme on iodine enriched eggs in the form of biological active iodo-organic compounds from animal origins was started by selecting two neighbourhood villages in those base-line study areas: Ban Nong Nok Kean and Ban Kew in Napu sub-district of Udon Thani province. A hen egg farm located in these two village areas was selected as a model farm for the supply of iodine enriched eggs to the communities. The base-line of iodine content in eggs from regular feeding at this model farm was done by collecting 30 fresh eggs weight between 50-55 g, and determining the iodine content according to the TCM-040 based on Compendium of Methods for Food Analysis [9]. Then, the programme was initiated by replacing regular feed with the iodine enriched feeding formula prepared by the addition of iodine as potassium iodide (KI) to yield a final concentration of 4 mg of iodine per kilogram of poultry feed for the daily feeding process of the farm. The hens consumed the iodine poultry feed at the amount of 120-130 g per hen per day. After one month feeding, 30 eggs were collected and sent to the laboratory for the determination of iodine content.

The biological active form of iodine from the iodine enriched eggs was evaluated by the determination of urine iodine excretion of volunteers before and after consuming a hard boiled iodine enriched egg continuously for five days, as one item in their breakfast and no other iodine enriched food was taken in the volunteers' meals during these five days. The first morning mid-stream urine from each volunteer was collected and the urine specimens were kept frozen at -20°C until the analysis. There were 124 women volunteers from these two villages, age between 20 - 63 years. All village volunteers were explained about the study details and consent forms were given to the volunteers for their agreement for study participation with an included statement that they could dropped out from this study at any time. The iodine content in urine was determined using the ICP-MS procedure. The median urine iodine was calculated and ANOVA was used for statistic analysis of the urine samples from the village volunteers for the treatment before and after eating iodine enriched eggs.

## Results

Table 1 showed that in August 2006 the median urine iodine of 858 child bearing-age women volunteers was 5.16 μg/dL (range from 3.26 - 7.68 μg/dL in all 5 districts) which meant that the villagers had an iodine deficiency condition even though the campaign from the provincial health office and district health centers emphasized their public health education programme on the use of iodized salt.

**Table 1:** Median urine iodine contents from child bearing-age women in the study areas of Udon Thani province in August 2006

Province	District	Sub-district	Village	No. of samples	Median Urine Iodine (μg/dL)
Udon Thani	Nam Som	Nam Som		**101**	**6.50**

			Ban Phon	69	7.68
			
			Ban Na Muang Thai	32	3.86

Udon Thani	Ban Dung			**174**	**4.97**

		Ban Chantr	Ban Subsomboon	59	3.26
		
		Ban Chai	Ban Non Sa-ard	55	4.96
		
		Srisutho	Ban Sriburapa	60	6.45

Udon Thani	Pen	Napu		**128**	**4.61**

			Ban Kew	63	3.82
			
			Ban Nong Nok Khaen	65	5.24

Udon Thani	Muang			**212**	**6.14**

		Ban Jan	Ban Dong Keng	73	7.09
		
		Sam Praw	Ban Na Yaad	66	6.26
		
		Nong Na Kham	Ban Jampa	73	4.97

Udon Thani	Sri That	Tad Thong		**238**	**4.64**

			Ban Kud Na Khor	74	5.01
			
			Ban Pa Whai	67	4.67
			
			Ban Ratsomboon	97	4.07

**Total in Udon Thani province**				**858**	**5.16**

Table 2 showed the iodine content of hen eggs produced by the model farm. It was found that the average iodine content in eggs from the regular feeding formula was 25.31 μg per egg (or 75.96 μg per 100 gram of fresh weight) whereas the iodine enriched feeding formula yielded the mean iodine content in the range of 93.57 - 97.76 μg per egg (or 182.67 - 184.58 μg per 100 gram of fresh weight). The production batches that was provided to the village volunteers for their breakfast had an iodine content in the range of 90.97 - 104.14 μg per egg for egg size #2 (60 - 65 g per egg), and a range of 87.76 - 98.18 μg per egg for egg size #3 (55 - 60 g per egg).

**Table 2:** Iodine content in eggs from the experimental farm before and after the iodine enriched feeding

	Average iodine content in eggs	
	
	μg per egg	μg per 100 g.wt
Eggs from regular feeding (#4; 50-55 g)	25.31	75.96

Iodine enriched eggs (#3; 55-60 g)	93.57	182.67

Iodine enriched eggs (#2; 60-65 g)	97.76	184.58

Table 3 showed the level of urine iodine of the village volunteers. It was found that the base-line median urine iodine before consuming the iodine enriched eggs from 65 volunteers in Ban Nong Nok Kean was 7.04 μg/dL (SD = 8.54, n = 65), and from 59 volunteers in Ban Kew was 7.00 μg/dL (SD = 7.15, n = 59). The median urine iodine from 124 volunteers in these two villages was 7.03 μg/dL (SD = 7.89, n = 124) which indicated the mild iodine deficiency condition of these two village areas.

**Table 3:** Urine iodine contents of women volunteers in Ban Nong Nok Kean village and Ban Kew village before consuming iodine enriched eggs

Urine iodine content (μg/dL)	Ban Kew village	Ban Nong Nok Kean village	Total (2 villages)
maximum	37.30	45.51	45.51

minimum	1.00	1.51	1.00

Std.Dev	7.15	8.54	7.89

Median	7.00	7.04	7.03

Population of volunteers	59	65	124

Table 4 showed the results for urine iodine levels of the village volunteers after consuming a hard boiled iodine enriched egg continuously for five days as one item in their breakfast meals. The result of a median urine iodine from 55 volunteers of Ban Nong Nok Kean was 13.95 μg/dL (SD = 10.76, n = 55), and from 57 volunteers of Ban Kew was 20.76 μg/dL (SD = 13.63, n = 57). The median urine iodine from the volunteers in these two villages after having iodine enriched eggs was 16.57 μg/dL (SD = 12.56, n = 112).

**Table 4:** Urine iodine contents of volunteers in Ban Nong Nok Kean village and Ban Kew village after consuming iodine enriched eggs

Urine iodine content (μg/dL)	Ban Kew village	Ban Nong Nok Kean village	Total (2 villages)
maximum	61.2	50.93	61.2

minimum	3.94	3.53	3.53

Std.Dev	13.63	10.76	12.56

Median	20.76	13.95	16.57

Number of volunteers	57	55	112

Table 5 demonstrated the comparison using statistic ANOVA analysis of urine iodine content of the women volunteers from the two villages in the study: before and after consuming iodine enriched eggs. The result showed that there was a highly significant difference (P value < 0.001) in the urine iodine content of volunteers before and after consuming iodine enriched eggs.

**Table 5:** Comparison of urine iodine contents of women volunteers (as the same volunteers) with treatment before and after consuming iodine enriched eggs in Ban Nong Nok Kean village and Ban Kew village

	Median urine iodine contents (μg/dL)	
	
	Ban Kew village	Ban Nong Nok Kean village
Before consuming iodine enriched eggs	6.87	7.11

After consuming iodine enriched eggs	20.76	13.09

F-test	P < 0.001	P < 0.001

Statistical signification	**	**

Number of volunteers	57	53

** highly statistical significant as P value < 0.001

Note: There were 14 volunteers (12 in Ban Nong Nok Kean village and 2 in Ban Kew village) that dropped out during the study.

## Discussion

Before the implementation of the iodine enrichment programme in the natural food chain of study areas, the median urine iodine from 858 volunteers (Table 1) in 5 districts of Udon Thani province was 5.16 μg/dL (range 3.26 - 7.68 μg/dL). This demonstrated that the community urine iodine level was at a mild iodine deficiency status [10], even though there was an active campaign in the region to encourage consumption of edible iodized salts by the provincial health office.

The iodine enriched feeding formula was successfully prepared and yielded about 3.8 fold higher iodine content in the produced eggs than the regular formula (Table 2). Thus, the iodine enriched feeding formula could be used instead of the regular one and the cost of iodine added in the formula was only 0.33% of the cost per kg of poultry feed. This would not significantly affect the cost of hen egg production.

The result of urine iodine survey before the dietary enrichment of iodine diet in two villages of the study area: Ban Kew village and Ban Nong Nok Kean village in Napu sub-district, Udon Thani province was 7.03 μg/dL (Table 3) which confirmed that these two communities were still in the condition of mild iodine deficiency. These results also showed that during the period of 2006 - 2008 the diets consumed by the community were still lacking iodine and the concerned public health sectors had not created any awareness to eliminate this iodine deficiency crisis.

For the five day study of continuously consuming an iodine enriched egg as one item at breakfast, there were 124 volunteers that participated at the beginning of the study and 14 volunteers dropped out in later stage. There were 53 volunteers from Ban Nong Nok Kean village and 57 volunteers from Ban Kew village that participated through out the study programme. By using an ANOVA method for statistical assessment, the result showed that the median urine iodine level before and after consumption of iodine enriched eggs were highly significant different at P < 0.001 (Table 5). The effect of this innovative process produced a remarkable increase of the urine iodine level from the condition of iodine deficiency (median urine iodine content 6.87 - 7.11 μg/dL) to the optimal level of iodine (median urine iodine content 13.09 - 20.76 μg/dL) in the villagers of the study areas. The urinary iodine excretion could also be used as a valid marker for reporting the recent dietary iodine intake as well as one of the key indicators for monitoring the IDD situation at the community level and the sustained adherence to public health efforts to eliminated IDD.

## Conclusions

In summary, the pilot model farm for the production of iodine enriched eggs supplied to the neighbouring communities was successfully developed which resulted in a self-support system of nutritional iodine enrichment for the communities. The WHO/UNICEF/ICCIDD has recommended that the daily iodine in take for various age groups be in the range between 90 - 200 μg. Since eggs could be consumed as daily food products in every Thai family, it would be possible to supply iodine eggs as the new iodine daily diet source to all Thai communities due to the fact that the cost for the addition of iodine as potassium iodide or potassium iodate to poultry feed was very low and did not significant increase the production cost of eggs from the farm. In addition, the preparation of iodine enriched formula poultry feed could be self processed at the farms and did not require any complicated equipments for the iodine enrichment steps. This innovative and inexpensive strategy could be easily applied to all remote areas throughout the country with the community programme of Sufficient Economy Concept to overcome the problem of iodine deficiency endemic in Thailand.

## Competing interests

The authors declare that they have no competing interests.

## Authors' contributions

WC was responsible for the conception and the design of the model programme, interpretation of data, field works, drafting and approval of the manuscript. PS participated in design the programme on the aspect of iodine enrichment for animal feeds, field works, data analysis and drafting the manuscript. PT participated in the analysis of urine samples by ICPMS. JW participated in field works and specimens collection. All authors read and approved the final manuscript.
